# Effectiveness of Different Algorithms and Cut-off Value in Preeclampsia First Trimester Screening

**DOI:** 10.1155/2022/6414857

**Published:** 2022-04-08

**Authors:** Piotr Tousty, Bartosz Czuba, Dariusz Borowski, Magda Fraszczyk-Tousty, Sylwia Dzidek, Ewa Kwiatkowska, Aneta Cymbaluk-Płoska, Andrzej Torbé, Sebastian Kwiatkowski

**Affiliations:** ^1^Department of Gynecology and Obstetrics, Pomeranian Medical University, 70-111 Szczecin, Poland; ^2^Department of Obstetrics and Gynecology in Ruda Slaska, Medical University of Silesia, 41-703 Ruda Slaska, Poland; ^3^Clinic of Fetal-Maternal, Gynecology and Neonatolgy, Collegium Medicum, Nicolaus Copernicus University in Bydgoszcz, 85-821 Bydgoszcz, Poland; ^4^Department of Neonatal Diseases, Pomeranian Medical University, 70-111 Szczecin, Poland; ^5^Department of Nephrology, Transplantology and Internal Medicine, Pomeranian Medical University, 70-111 Szczecin, Poland; ^6^Department of Gynecological Surgery and Gynecological Oncology of Adults and Adolescents, Pomeranian Medical University, 70-111 Szczecin, Poland

## Abstract

**Results:**

For the cut-off point >1 : 150, 86 women at an increased risk of eo-PE using algorithm 1 were identified. Of these 86 patients, 83 (96%) were identified using algorithm 2, 62 (72%) using algorithm 3, and 60 (69%) using algorithm 4. In addition, it was demonstrated that between 21% and 29% of women at a low risk of eo-PE could be given acetylsalicylic acid if a screening test was used that did not account for PlGF.

**Conclusions:**

In order to provide the highest level of health care to pregnant women, it is extremely important that full screening for eo-PE should be ensured. The cheapest algorithm based only on MAP and UtPI resulted in our patients being unnecessarily exposed to complications.

## 1. Introduction

Preeclampsia (PE) is a multi-factorial disorder affecting 2% to 8% of pregnancies. Worldwide, it is one of the most important causes of maternal and fetal deaths, preterm labor, and hospitalizations in pathology of pregnancy departments and neonatal intensive care units [1, 2].

It has been found that women with a history of preeclampsia have a higher risk of developing ischemic heart disease, arterial hypertension, and thromboembolic disease, as well as other cardiovascular diseases in later life [3].

In recent years, developments in prenatal diagnosis have allowed prediction of preeclampsia. It has been shown that patients at risk of developing PE have different values of some of their biophysical and biochemical parameters as early as the first trimester. Examples of such parameters are the biochemical factors placental growth factor (PlGF) and pregnancy-associated plasma protein A (PAPP-A). In cases with threatened preeclampsia, PlGF and PAPP-A levels are reduced in the first trimester [4–9]. Another important parameter in assessing the risk of preeclampsia is the uterine artery pulsatility index (UtA-PI) in the first trimester ultrasound. Under normal conditions, UtA-PI decreases as pregnancy continues as a result of the remodeling of the spiral arteries and a decrease in their resistance. However, in the case of the risk of preeclampsia and, for example, FGR (Fetal Growth Restriction), the first trimester UtA-PI value is increased [10].

It has been noticed that the measurement of mean arterial pressure (MAP), as well, is important in the prediction of preeclampsia. In the physiological pregnancy, blood pressure decreases during the first and second trimesters, gradually returning to its pre-pregnancy values at the end of gestation and after delivery. However, in preeclamptic women, MA*P* values in the first and second trimesters are increased [11]. This new group of patients is identified through a comprehensive assessment of these parameters combined with maternal history, which together are an extremely effective predictor of PE, especially its early-onset form (before the 34th week of gestation (or wkGA)) (eo-PE) [4–11].

Unfortunately, there is currently no treatment available that would significantly extend the duration of gestation after a PE diagnosis. However, for women with an increased risk of eo-PE identified in the first trimester, acetylsalicylic acid (ASA) has been shown to reduce the incidence of preeclampsia prior to 34 wkGA by 82% compared to the placebo group. Furthermore, if the study had excluded women with chronic arterial hypertension and those that took less than 90% of the recommended doses, the risk of eo-PE would have fallen by 95% [12, 13].

There is a need for a continuous discussion on, and for doctors to be reminded of, the benefits of screening in pregnancy in order to better care for the pregnant patient and her child. The first aim of our study was to assess the detectability and the parameters of women at risk of developing eo-PE depending on the algorithm used. The second objective was to observe changes in the size of the groups taking acetylsalicylic acid depending on the cut-off point for an increased risk of eo-PE chosen and the algorithm used to detect the eo-PE risk group patients.

## 2. Patients and Methods

The prospective study conducted in 2019 included a population of 801 pregnant Caucasian patients from the Prenatal Testing Outpatient Clinics in Szczecin and Katowice as part of first trimester pregnancy screening tests (at 11–14 wkGA) in order to detect aneuploidy, fetal defects, and the risk of preeclampsia. The study was conducted in accordance with the Fetal Medicine Foundation (FMF) principles for the detection of women at risk of PE. The study was conducted with the consent of the bioethical committee at the Pomeranian Medical University in Szczecin (consent no. KB-0012/157/18). Each woman gave her written consent to participate in the study. Each patient's medical history was acquired, maternal characteristics were established (including their age, weight, height, parity, race, smoking history, diabetes mellitus type 1 or 2, chronic hypertension, systemic lupus erythematosus (SLE), antiphospholipid syndrome (APS), family history of preeclampsia, and the method of conception). Arterial pressure was measured using an automated blood pressure monitoring device twice per arm. A transabdominal probe of the Voluson E6 ultrasound system was used to measure the uterine artery pulsatility index (UtA-PI). The pulsatility index was determined for both uterine arteries, and an average value was calculated. Subsequently, blood samples were collected from each patient for PAPP-A and PlGF determinations. In Szczecin, the Cobas e 801 (Roche Diagnostics) analyzer was used to measure PlGF and PAPP-A. In Katowice, in turn, these parameters were measured using the DELFIA Xpress system (PerkinElmer Life). Subsequently, the biochemical parameter values were expressed in the MoM (a multiple of the median). Each patient was individually evaluated in terms of her risk of eo-PE based on the FMF algorithms (FMF -2012 software version 2.8.1). The patients were divided into four groups depending on the eo-PE risk calculation algorithm used:

1. screening based on UtA-PI, MAP, PlGF
2. screening based on UtA-PI, MAP, PAPP-A, PlGF
3. screening based on UtA-PI, MAP, PAPP-A
4. screening based on UtA-PI, MAP

According to the FMF and FIGO recommendations, the Maternal History+MAP+UtA-PI+PlGF algorithm is recommended for detecting women in the eo-PE risk groups for prophylaxis with 150 mg of ASA with the highest detection rate. We deemed this algorithm to be the most favorable and used it to compare with the other algorithms. We selected 3 cut-off points for the eo-PE risk groups to whom acetylsalicylic acid should be administered:

1. >1 : 150 – concordant with the recommendations of the Polish Society of Gynecologists and Obstetricians (PTGiP) and mentioned as a cut-off point that is suitable for the Caucasian population according to FMF
2. >1 : 100 – according to the FIGO recommendations
3. >1 : 70 – our own cut-off point for the group with the highest risk of PE

## 3. Statistical Analysis

The results of the study were statistically analyzed. The non-parametric Mann–Whitney *U*-test was used to calculate the differences in the tested parameters, and McNemar's test was used for the analysis of differences in the sizes of the individual groups. The Statistica ver. 13 software was used for the analysis (StatSoft, Poland).

## 4. Results

In our study, the FMF clinical algorithms were compared in order to detect women in the eo-PE risk groups.  Table 1 shows the general characteristics of the total population studied. Tables 2–4 show the differences in the parameters studied during the first trimester of pregnancy depending on the cut-off point and the algorithm used to calculate the risk of developing PE. We found no statistically significant differences among the cut-off points>1 : 70, > 1 : 100, and>1 : 150. The main reason for the lack of differences is the fact that, as shown in Table 5, some of the women were classified as high risk for eo-PE when different algorithms were used simultaneously. Therefore, when comparing differences between the groups (Tables 2–4), most were found to contain the same numbers of women, which resulted in a lack of statistical significance. This, however, came as no surprise to us, especially that exploring the differences between the parameters was not the primary aim of the paper. The most important objective was to show how many women would be prescribed ASA depending on the algorithm used and how many would not be administered ASA if we were not to use the algorithm accounting for PlGF determination.

Table 6 shows the numbers of the cases detected in the PE risk groups. As can be seen, the screen positive ratios (SPRs) for all the algorithms for the same cut-off points were similar. Clearly, as well, the algorithm including a PlGF determination alone (algorithm 1) did not differ significantly in the number of cases detected from the algorithm including PlGF and PAPP-A (algorithm 2). The same is confirmed by Table 5, where the algorithm including PAPP-A and PlGF for any cut-off point did not detect, in a statistically significant manner, fewer cases than the algorithm including PlGF alone.

As shown in Table 6, algorithm 1 had a 5.5% SPR for the risk cut-off point >1 : 70, 7.2% SPR for the risk cut-off point >1 : 100, and a 10.7% SPR for the risk cut-off point >1 : 150. According to Tables 5 and 6, a comparison of algorithm 1 with the other algorithms for the risk cut-off value >1 : 100 shows that algorithm 3 containing only PAPP-A detected 72.5% (42/58) of women in the high PE risk group, while algorithm 4 without the biochemical markers (History+MAP+UtPI) for the same cut-off value detected 71% (41/58) patients in the PE risk group. For the >1 : 150 group, these values were 72% (62/86) for algorithm 3 with PAPP-A and 70% (60/86) for algorithm 4 without the biochemical markers, respectively. These differences are confirmed by the data shown in Table 5, where in addition to a lack of statistical significance for the comparison of the algorithm including PlGF (algorithm 1) with the algorithm including PAPP-A (algorithm 3) for the cut-off point >1 : 70, all the others did actually demonstrate such significance. Table 5 shows the superiority of the algorithm containing PlGF over the algorithms that excluded it. In other words, algorithm 1 (screening based on UtA-PI, MAP, and PlGF) detected statistically significantly more women at risk of developing PE. For instance, with the cut-off point >1 : 150, algorithm 1 (which accounts for PlGF) has a statistically significant higher detection rate of high-risk women than other algorithms which do not include PlGF determination. For the cut-off point >1 : 100, these values were, respectively, 0.0001 compared with algorithm 4 (without biochemical parameters), and 0.0002 compared with the algorithm accounting only for PAPP-A.

Additionally, the data contained in Table 6 shows that while using algorithm 3 including PAPP-A to calculate the risk of PE for the cut-off point >1 : 70, an additional 10 out of 44 women (22%) who should be on ASA, considering algorithm 1 as the most significant, were deemed to be receiving it without reasonable grounds. For cut-off points >1 : 100 and >1 : 150, these proportions were 17/58 (29%) and 18/86 (21%), respectively. Similar relationships can be seen for algorithm 4, where for the cut-off points >1 : 70, > 1: 100, and >1: 150, these proportions were 10/44 (22%), 20/58 (34%), and 24/86 (28%), respectively. In other words, between 21% and 34%, more women were classified as high-risk patients, for whom ASA administration was recommended as long as the algorithm used did not account for PlGF.

## 5. Discussion

Proving the effect of acetylsalicylic acid on the incidence of preeclampsia among women in the risk groups has been one of the greatest achievements in obstetrics and gynecology of recent years. It is particularly worth recalling the falling incidence of early-onset PE, i.e., <34 wkGA, which is after all responsible for most neonatal complications. In the ASPRE study, patients with a PE risk of >1 : 100 according to the FMF algorithm were assumed to be included in the risk groups [12, 13].

However, the choice of the appropriate cut-off point and indications for using ASA is still a controversial subject discussed in various societies, as research continues. According to the ACOG and NICE, it is sufficient if the relevant criteria are met without considering the biophysical and biochemical factors, reaching different DRs at the same time: 94% and 41%, respectively, for eo-PE. In the first case, unfortunately, despite the high DR, the FPR reached values exceeding 60%. The most accurate screening model as of today is the one proposed by the FMF, which for a relatively low FPR of 10% gives, according to various reports, a DR of approx. 90% for eo-PE. Therefore, the aim of the present study was to examine the different algorithms proposed by FMF and compare them at different cut-off point levels for the high-risk group [4, 6, 8, 14–16].

The FIGO, The FMF, and the Polish Society of Gynecologists and Obstetricians recommend using the FMF algorithm including maternal history, MAP, UtA-PI, and PlGF, since it has the highest predictive value with the cut-off points >1 : 100 (similar to the FIGO recommendations and >1 : 150 (similar to the FMF and Polish Society of Gynecologists and Obstetricians (PTGiP) recommendations). The different cut-off points are selected in relation to the characteristics of the selected population. For the Caucasian population, as in our study, the most appropriate cut-off point is that proposed by the FMF and the PTGiP because, as research demonstrates, the DR is 80-94% for eo-PE at an SPR of approx. 15%. In the present study, the SPR for the cut-off point of 1 : 150 was 10.7%, meaning it was in line with the model proposed by the FMF. The authors show that a more conservative choice of risk groups, i.e., as FIGO suggests - >1 : 100 or even >1 : 70, may fail to achieve the desired DR, especially for the Caucasian population. In contrast, it should be remembered that the DR for the cut-off point >1 : 150 for the Afro-Caribbean population was 100% at a 40% FPR, and that is why the researchers established a more conservative approach for this population [4, 8, 16–19].

Wishing to help our patients as much as possible, we strive to detect pathologies as early as practicable. Clearly, the FMF algorithm including PlGF that we studied proved to be the best method for detecting the risk. Using other FMF algorithms, or adopting the approaches recommended by associations such as the ACOG or the NICE, a large proportion of women are caused to receive ASA despite being at no risk of developing eo-PE. In our study, for the cut-off point >1 : 150, 21-28% of the women received ASA while actually belonging to the low-risk group if no PlGF was included in the screening. Similarly, for the cut-off point >1 : 100, these numbers were between 29 and 34%, and for the cut-off point >1 : 70, the calculated value was 22% [4, 15, 16, 19, 20].

There are discussions pending on whether or not acetylsalicylic acid should be made available to all pregnant patients equally, regardless of the risk group they belong to [21]. Nevertheless, it still appears reasonable that the smaller the amounts of drugs administered to pregnant women the better. In addition, many of these patients would not be willing to accept acetylsalicylic acid if no indications were observed in them. To our knowledge, no randomized studies are available at present assessing the long-term safety of using ASA in all pregnant women. Of note, there are reports in the literature that ASA may increase the risk of vaginal bleeding during pregnancy, as well as gastroschisis or cerebral palsy [22–24]. Gastroschisis, however, is caused when ASA is administered in the first trimester, i.e., theoretically before the point of less than 16 wkGA recommended for the inclusion of ASA in the management of women at high risk of eo-PE [22]. At the same time, a study showing an increased risk of cerebral palsy does not propose an ASA dose, while other authors show that there is no such relationship, although their study was performed on a much smaller group of patients [23]. Nevertheless, as we mentioned, our study shows that up to an additional 34% of women can be given ASA if we do not use PlGF in our eo-PE risk calculation.

Screening with PlGF shows a high-risk pregnancy group. In clinical practice, it is extremely important, what percentage of whole pregnant population will be treated as high-risk pregnancies, in relation to women, who will achieve measurable benefits. If we use the most precise algorithms, we will achieve definitely much better results. At the present time, PAPP-A tests are quite common, despite the relatively high effectiveness, the PlGF algorithms are characterized by higher efficiency [5–9, 17, 18].

Our results confirm previous reports that adding PAPP-A to the algorithm recommended by the FIGO, the FMF and the PTGiP does not change screening effectiveness in detecting women at risk of eo-PE. [19] For none of the cut-off points was the difference between the two algorithms statistically significant.

On the other hand, we showed a statistically significant superiority of algorithm 1 (with PlGF) over the popular algorithm (only with PAPP-A = algorithm 3). In our opinion, it is a dangerous phenomenon whereby an increasing number of women is being classified in the high-risk pregnancy groups. The use of PlGF helps to mitigate this tendency.

When talking about the impact of aspirin on PE, other forms of the condition must be addressed, as well. As shown by the ASPRE study, for instance, aspirin reduces the risk of eo-PE and preterm PE occurring prior to 37 wkGA. This, however, does not apply to other forms of PE, especially its term variants. As the authors implicate, this may be caused by a number of factors. Firstly, if administered early enough (prior to 16 wkGA), aspirin assists in spiral artery remodeling, thus deepening placentation. This brings about a reduction in the overall incidence of the more severe eo-PE, or perhaps simply defers the time of its occurrence for the benefit of late-onset PE or term PE, which is milder. Secondly, the causes of term PE are often not related to impaired spiral artery remodeling but are associated with maternal predispositions and co-morbidities, such as chronic arterial hypertension or kidney diseases, which lead to vascular endothelial dysfunction. In these cases, aspirin will not reduce the incidence of PE. At the same time, it should be noted that in term PE cases, the perinatal outcomes are usually good, and the treatment focuses primarily on the mother [13, 24–27].

Another important aspect is compliance with the aspirin regimen if we were to qualify most of the population for ASA use. Previous studies in some ways are of disagreement over qualifying different proportions of the population for ASA use. For example, the ASPRE study (qualifying women for ASA use based on algorithm 1 from our study with cut-off point >1 : 100) shows that in women who do not have chronic hypertension and whose adherence rate is >90%, the eo-PE frequency will drop by approximately 90% [12]. Another study shows that the indiscriminate use of aspirin (i.e., in all pregnant patients) may lead to an even greater reduction in the incidence of both eo-PR and lo-PE than if ASA were to be only administered to the high-risk women singled out using the algorithm that accounts for PlGF determination. However, this study assumes a compliance level of 100%, which is almost impossible to achieve [28]. Similar to the results of other studies, lower compliance levels (<90%), which in our opinion are more realistic, do not lead to such reductions in PE incidence [29, 30]. In our assessment, it is necessary for each patient to be offered a screening test for PE using the best possible methods in line with the EBM guidelines in order to minimize the risk of serious complications occurring in them or their children [5, 6, 9]. Our study shows that we detect only 70–72.5% of women at risk of developing eo-PE if we do not use the PlGF algorithm (algorithms 1 or 2) to calculate the risk of its occurrence. There is no doubt that the cost and the low availability of screening tests accounting for PlGF are factors limiting their common application. For a large proportion of women, the cost charged by private health care facilities may be too high [15].

This does not prevent the conclusion that, for the public health care system, complete screening tests for the risk of eo-PE accounting for PlGF, and an appropriate qualification for ASA treatment, will significantly reduce the overall cost of prenatal. This is confirmed by the ASPRE study, where newborns from mothers treated with ASA had significantly shorter hospitalizations in the neonatal intensive care unit (NICU) [31]. Another study carried out in Canada shows that subjecting all pregnant patients to complete screening tests accounting for PlGF combined with an appropriate qualification for ASA treatment will result in approx. C$14 million (€9.5 million) in annual savings for the health care system [32].

It may also be necessary to monitor women from groups at risk of developing PE. Women with a history of PE are known to be at risk of cardiovascular events in the future. The question is whether or not the women from these risk groups demonstrate an increased risk of developing such conditions, as well [3]. Perhaps, large randomized trials would be able to assess this issue.

Our study is a reminder to doctors that every woman should be offered risk stratification for the development of eo-PE. Cheaper screening tests, with no PlGF determination, expose our patients to complications. Many doctors, also in Poland, only offer prenatal diagnosis of the risk of aneuploidy including tests for *β*-hCG (B-human chorionic gonadotropin) and PAPP-A. This examination is undoubtedly an important part of prenatal diagnosis and care during pregnancy. However, it should be borne in mind that we are currently operating within a care model that puts emphasis on early detection of risks, while PE is one of the most serious risks in pregnancy.

## 6. Conclusions

The risk of a pregnant patient developing eo-PE should be assessed using the FMF algorithm that accounts for their medical history, maternal characteristics, MAP, UtA-Pl, and PlGF. Applying other algorithm types results in unnecessary ASA administration to some patients on the one hand, and failure to administer it to some patients carrying an increased risk on the other.

## Figures and Tables

**Table 1:** Characteristics of the study group (CRL: crown rump length; SLE: systemic lupus erythematosus; APS: Antiphospholipid syndrome).

	Median (IQR)		*n* (%)
Age	32 (27-35)	Smoking	41 (5.1)
Weight	65 (58-74)	Diabetes mellitus type 1	11 (1.37)
Height	165 (162-170)	Diabetes mellitus type 2	8 (1)
Parity	1 (0-2)	Chronic hypertension	25 (3.12)
CRL	64.1 (59.4-68.7)	SLE/APS	8 (1)
MoM UtPI	1.1 (0.9-1.32)	Nulliparous	254 (31.7)
MoM PAPP-A	1.04 (0.68-1.39)	Parous previous PE	26 (4.75)
MoM PlGF	0.96 (0.73-1.25)	Family history of PE	19 (2.37)
MoM MAP	1.04 (0.98-1.11)	In vitro fertilization	6 (0.75)

**Table 2:** Differences between first trimester screening parameters according to the algorithm with a cut-off point for a PE risk >1 : 70 (n: number of patients; CRL: crown rump length; GA: gestational age (weeks); IQR: interquartile range).

Cut-off >1 : 70					
Type of algorithm	(1) History+MAP+UtPI+PlGF	(2) History+MAP+UtPI+PlGF+PAPP-A	(3) History+MAP+UtPI+PAPP-A	(4) History+MAP+UtPI	*p* value
Screen positive rate *n* (%)	44/801 (5.5)	47/801 (5.9)	51/801 (6.4)	48/801 (6)	
Age median (IQR)	35 (28.5-37)	35 (28-37)	35 (28-37)	44.5 (27.5-37)	0.96
Weight median (IQR)	73.25 (63-90)	72 (63-89)	72 (62.5-89)	74.75 (63.5-89)	0.97
Height median (IQR)	164 (160-169)	164 (160-170)	165 (160-170)	164.5 (160.5-170)	0.98
Parity median (IQR)	1 (0-1.5)	1 (0-1)	1 (0-2)	1 (0-2)	0.97
CRL(GA) median (IQR)	12.86 (12.43-13)	12.86 (12.43-13)	12.71 (12.29-13)	12.86 (12.57-13)	0.88
MoM UtPI median (IQR)	1.41 (1.12-1.58)	1.39 (1.12-1.58)	1.39 (1.12-1.58)	1.42 (1.13-1.59)	0.95
MoM PAPP-A median (IQR)	0.98 (0.7-1.27)	0.98 (0.69-1.29)	0.89 (0.68-1.26)	0.98 (0.7-1.26)	0.9
MoM PlGF median (IQR)	0.96 (0.8-1.19)	0.93 (0.74-1.17)	0.93 (0.76-1.2)	0.97 (0.8-1.19)	0.97
MoM MAP median (IQR)	1.23 (1.14-1.28)	1.22 (1.11-1.28)	1.22 (1.11-1.26)	1.17 (1.11-1.26)	0.49

**Table 3:** Differences between first trimester screening parameters according to the algorithm used with a cut-off point for a PE risk >1 : 100 (n: number of patients; CRL: crown rump length; GA: gestational age(weeks); IQR: interquartile range).

Cut-off >1 : 100					
Type of algorithm	(1) History+MAP+UtPI+PlGF	(2) History+MAP+UtPI+PlGF+PAPP-A	(3) History+MAP+UtPI+PAPP-A	(4) History+MAP+UtPI	*p* value
Screen positive rate *n* (%)	58/801 (7.2)	60/801 (7.5)	59/801 (7.36)	61/801 (7.6)	
Age median (IQR)	34.5 (28-37)	34.5 (28-36.5)	34 (28-36)	34 (28-36)	0.81
Weight median (IQR)	74.25 (63-89)	72 (62.75-89)	71 (61-89)	71.8 (62.5-89)	0.88
Height median (IQR)	164 (161-168)	164 (160.5-168)	165 (160-170)	165 (161-170)	0.81
Parity median (IQR)	1 (0-2)	1 (0-1.5)	1 (0-2)	1 (0-2)	0.96
CRL(GA) median (IQR)	12.86 (12.43-13)	12.86 (12.29-13)	12.71 (12.29-13)	12.86 (12.43-13)	0.92
MoM UtPI median (IQR)	1.34 (1.13-1.49)	1.32 (1.1-1.49)	1.36 (1.13-1.58)	1.39 (1.14-1.57)	0.93
MoM PAPP-A median (IQR)	0.98 (0.69-1.29)	0.95 (0.68-1.27)	0.95 (0.68-1.26)	0.94 (0.69-1.26)	0.98
MoM PlGF median (IQR)	0.97 (0.8-1.21)	0.93 (0.69-1.19)	0.95 (0.75-1.19)	0.97 (0.76-1.2)	0.87
MoM MAP median (IQR)	1.19 (1.09-1.26)	1.19 (1.1-1.27)	1.19 (1.11-1.26)	1.18 (1.11-1.25)	0.98

**Table 4:** Differences between first trimester screening parameters according to the algorithm used with a cut-off point for a PE risk >1 : 150 (n: number of patients; CRL: crown rump length; GA: gestational age(weeks); IQR: interquartile range).

Cut-off >1 : 150					
Type of algorithm	(1) History+MAP+UtPI+PlGF	(2) History+MAP+UtPI+PlGF+PAPP-A	(3) History+MAP+UtPI+PAPP-A	(4) History+MAP+UtPI	*p* value
Screen positive rate *n* (%)	86/801 (10.7)	87/801 (10.9)	80/801 (10)	84/801 (10.5)	
Age median (IQR)	33 (28-36)	33 (28-36)	32 (27.5-36)	33 (28-36)	0.9
Weight median (IQR)	71 (62.5-85)	70 (62-85)	69.1 (60.5-84)	70.5 (62.25-85.5)	0.96
Height median (IQR)	164 (160-168)	164 (160-168)	165 (160-170)	165 (160-170)	0.86
Parity median (IQR)	1 (0-2)	1 (0-1)	1 (0-1.5)	1 (0-1)	0.96
CRL(GA) median (IQR)	12.71 (12.29-13)	12.71 (12.29-13)	12.71 (12.29-13)	12.71 (12.29-13)	0.96
MoM UtPI median (IQR)	1.35 (1.13-1.55)	1.34 (1.13-.55)	1.35 (1.13-1.56)	1.34 (1.12-1.52)	0.97
MoM PAPP-A median (IQR)	0.96 (0.68-1.35)	0.98 (0.68-1.35)	0.96 (0.68-1.28)	0.94 (0.67-1.26)	0.89
MoM PlGF median (IQR)	0.93 (0.72-1.27)	0.94 (0.73-1.27)	0.93 (0.72-1.2)	0.92 (0.71-1.2)	0.92
MoM MAP median (IQR)	1.16 (1.08-1.25)	1.16 (1.09-1.25)	1.17 (1.11-1.25)	1.17 (1.11-1.25)	0.89

**Table 5:** Differences in the detectability of patients in the PE risk group using other algorithms compared to algorithm 1 (History+MAP+UtA-PI+PlGF).

Method of screening	Comparison of detection by two methods	*p* value
*Preeclampsia cut-off 1 : 70*		
History+MAP+UtPI+PlGF vs History+MAP+UtPI+PlGF+PAPP-A	44vs44	1.00
History+MAP+UtPI+PlGF vs History+MAP+UtPI+PAPP-A	44vs41	0.25
History+MAP+UtPI+PlGF vs History+MAP+UtPI	44vs38	0.04
*Preeclampsia cut-off 1 : 100*		
History+MAP+UtPI+PlGF vs History+MAP+UtPI+PlGF+PAPP-A	58vs56	0.48
History+MAP+UtPI+PlGF vs History+MAP+UtPI+PAPP-A	58vs42	0.0002
History+MAP+UtPI+PlGF vs History+MAP+UtPI	58vs41	0.0001
*Preeclampsia cut-off 1 : 150*		
History+MAP+UtPI+PlGF vs History+MAP+UtPI+PlGF+PAPP-A	86vs83	0.25
History+MAP+UtPI+PlGF vs History+MAP+UtPI+PAPP-A	86vs62	<0.0001
History+MAP+UtPI+PlGF vs History+MAP+UtPI	86vs60	<0.0001

**Table 6:** Characteristics of the groups in terms of the cases detected compared to algorithm 1 (History+MAP+UtA-PI+PlGF) (n: number of patients; ASA: acetylsalicylic acid).

		(1) History+MAP+UtPI+PlGF	(2) History+MAP+UtPI+PlGF+PAPP-A	(3) History+MAP+UtPI+PAPP-A	(4) History+MAP+UtPI
1 : 70 cut-off	Screen positive rate *n* (%)	44 (5.5)	47 (5.9)	51 (6.4)	48 (6)
	Cases found by both algorithm 1 and the tested algorithm *n* (%)		44 (5.5)	41 (5.1)	38 (4.7)
	Additional cases found for unnecessary ASA prophylaxis *n* (%)		3 (0.37)	10 (1.2)	10 (1.2)

1 : 100 cut-off	Screen positive rate *n* (%)	58 (7.2)	60 (7.5)	59 (7.36)	61 (7.6)
	Cases found by both algorithm 1 and the tested algorithm *n* (%)		56 (7)	42 (5.2)	41 (5.1)
	Additional cases found for unnecessary ASA prophylaxis *n* (%)		4 (0.5)	17 (2.1)	20 (2.5)

1 : 150 cut-off	Screen positive rate *n* (%)	86 (10.7)	87 (10.9)	80 (10)	84 (10.5)
	Cases found by both algorithm 1 and the tested algorithm *n* (%)		83 (10.4)	62 (7.8)	60 (7.5)
	Additional cases found for unnecessary ASA prophylaxis *n* (%)		4 (0.5)	18 (2.2)	24 (3)

## Data Availability

Data available on request. Data is in a form of tables including patient personal information. The corresponding author should be contacted to request the data.
